# A Case of COVID-19 Vaccine Causing a Myasthenia Gravis Crisis

**DOI:** 10.7759/cureus.15581

**Published:** 2021-06-10

**Authors:** Ariana R Tagliaferri, Spandana Narvaneni, Moh'd Hazem Azzam, William Grist

**Affiliations:** 1 Radiology, Sidney Kimmel Medical College at Thomas Jefferson University Hospital, Philadelphia, USA; 2 Internal Medicine, St. Joseph's Regional Medical Center, Paterson, USA; 3 Pulmonology and Critical Care, St. Joseph's Regional Medical Center, Paterson, USA

**Keywords:** covid-19, myasthenia gravis crisis, myasthenia gravis exacerbation, vaccination, moderna vaccine

## Abstract

Myasthenia gravis is a rare disease of the neuromuscular junction subsequently affecting the bulbar, respiratory, and extremity skeletal muscles. It is an autoimmune disease in which antibodies target the acetylcholine receptor (AChR), preventing transmission of the excitatory cascade during muscle contraction. Myasthenia gravis is typically well controlled using acetylcholinesterase inhibitors, steroids, immunosuppressant agents, and/or thymectomies. However, exacerbations can be induced by infection or medications. This is particularly important during the coronavirus disease 2019 (COVID-19) pandemic in which myasthenia gravis patients have been known to have poorer outcomes. We report a very rare presentation of a myasthenia gravis crisis induced by the Moderna COVID-19 vaccine.

## Introduction

Myasthenia gravis (MG) is a rare autoimmune disease that can become potentially serious [1]. It is the most common neuromuscular junction (NMJ) disorder characterized by antibodies against the acetylcholine receptor (AChR), which subsequently results in defective transmission of the polarization cascade in muscle contraction [1,2]. As the antibodies destroy the AChRs, efforts to contract the muscle exacerbate muscle weakness, although this can be improved with rest [1,2]. The bulbar, limb, and respiratory muscles can all be affected [1].

MG was first described by German doctors in 1895 as “pseudo-paralytica” [1]. MG has an incidence of approximately 0.04-5.0/100,000 per year and may affect any age group [1]. Previous studies have demonstrated a prevalence of 77.7 cases per million per year, and cases are rising as medical research and diagnostics are improving in the medical field [3]. It is not common for the onset of symptoms to appear in the first decade of life nor after the age of 70 years. Males are predominantly affected with ocular symptoms; however, the ratio of females and males affected by generalized myasthenia is 3:2 [1,3]. There are specific disease subtypes with distinct immune-pathogenic mechanisms [4]. Numerous studies have described decreased activation of B and T cells due to environmental factors, genetics, and aging [3,4].

The most common presenting complaints are ocular symptoms, with ptosis and diplopia present in over 50% of patients [1]. Other ocular symptoms can present as cranial nerve palsies or mimic strokes [1]. The extraocular muscles are affected first because their synapse fire at higher frequencies than limb muscles [1]. Within the first two years of symptom onset, patients will progress to generalized muscle weakness in over 90% of cases; patients who present with isolated ocular findings will progress to have generalized skeletal muscle weakness within two years after initial symptom onset [1]. Diagnosis can be made via clinical, laboratory, or neuromuscular testing. Clinical tests include the sleep test or ice test. The edrophonium test is 95% sensitive for generalized MG and allows clinicians to assess muscle strength and function before and after administration of a drug that prevents the breakdown and thus the release of ACh at the neuromuscular junction (NMJ) [1]. A similar drug with a longer mechanism of action is neostigmine [1]. Diagnosis can also be made with electrophysiological testing via repetitive nerve stimulation and single-fiber electromyography [2]. Treatment with physostigmine was first described in 1934 by Mary Walker [1]. Treatment for MG aims to alleviate symptoms of muscle weakness while slowing disease progression [1]. Current methods of managing MG include administration of acetylcholinesterase inhibitors such as pyridostigmine, corticosteroids, immunosuppressive therapy, plasmapheresis, intravenous immunoglobulin (IVIg), or thymectomy [1,2,4]. All patients are recommended to undergo computerized tomography (CT) imaging to rule out concurrent thymomas [1,2]; 15% of patients will develop thymomas further complicating their disease [1]. Older patients are more likely to experience a more severe form of MG with multiple relapses, higher complication rate, and poorer outcomes [3]. Any patient with comorbidities is also likely to experience exacerbations and worse side effects from medications [3]. Furthermore, patients with neuromuscular disorders and autoimmune diseases are at higher risk of not only acquiring coronavirus disease 2019 (COVID-19) during the pandemic but also of worse outcomes compared to healthy people [5-7]. Due to the relative immunocompromised state with superimposed respiratory and/or bulbar weakness, studies have shown that MG patients develop severe acute respiratory distress syndrome, disease exacerbations, further neurological complications, and have a higher mortality rate when hospitalized due to COVID-19 [5,6]. However, a relationship between the COVID-19 vaccine and MG exacerbations is yet to be established. We describe a rare case of an MG crisis induced by the COVID-19 vaccination.

## Case presentation

A 77-year-old Caucasian male with a past medical history of MG presented to the emergency room (ER) with complaints of dysphagia for one week. The patient was first diagnosed with MG five years ago and has been maintained on prednisone 7.5-milligram tablet daily and pyridostigmine 60-milligram tablet six times daily. The dysphagia was associated with non-specific arthralgia, intermittent fevers, chills, and fatigue. The remaining review of systems is negative. He is compliant with medications. He received the first and second doses of Moderna COVID-19 vaccine approximately five and one weeks prior to presentation. In the ER, vitals were notable for tachypnea (20 breaths per minute), tachycardia (110 beats per minute), and hypoxia (Pox 91% on room air). An electrocardiogram showed sinus tachycardia. Chest x-ray was negative for any infiltrates, effusions, or consolidations. Labs were significant for acute renal failure (due to poor oral intake). The septic workup was negative. He was admitted to the neuro floor with MG exacerbation. He was started on pyridostigmine 60 milligrams every four hours, prednisone 7.5 milligrams daily, and intravenous (IV) immunoglobulin (Ig) 35 grams daily for five days. His acute renal failure resolved with adequate IV hydration. Over the next few days, the patient's oxygen requirement normalized, but the dysphagia persisted. On day six of admission, he acutely decompensated with another crisis of MG manifested by lethargy and flaccidity. An arterial blood gas analysis (ABG) confirmed acute hypercapnic respiratory failure for which the patient was intubated and transferred to the medical intensive care unit (MICU). He was started on prednisone 10 mg (which was gradually increased in 48-hour intervals to 40 mg), another two IVIg were given on days 9 and 10. Serial negative inspiratory force (NIF) readings were obtained, with improvement from -20 to more than -40 and subsequent successful extubation. The MG crisis was attributed to the second dose of the Moderna COVID-19 vaccine.

## Discussion

MG exacerbations are commonly triggered by infections, especially during the COVID-19 pandemic [8]. Since the first outbreak, there have been numerous reports of COVID-19 causing more severe complications, including acute respiratory distress syndrome and worsening neurological symptoms in patients with MG [5-7]. When medications such as hydroxychloroquine and azithromycin were initially used to treat acute COVID infections, they were found to exacerbate MG [5,8]. One retrospective study observing MG patients among four hospitals in Brazil found that 87% of all MG-COVID patients were admitted to the MICU with 73% of those patients requiring mechanical ventilation and 30% of those patients who died [5].

Although numerous studies have observed the relationship between active COVID infections and MG exacerbations, no known studies or case reports have described an MG exacerbation induced by the COVID-19 vaccine. Our patient’s septic workup was negative, and he was compliant with his medications. Given the history of the COVID-19 vaccine coinciding with the onset of his dysphagia, it is most likely that our patient’s exacerbation was attributed to the vaccination. It is plausible that patients can acquire an infection as not only are the regulatory T and B cells in MG-defective but patients who are also commonly on immunosuppressive therapy [4]. There is also evidence to demonstrate a cytokine storm in COVID-MG patients, further exacerbating symptoms [7,9]. It is also then plausible that either our patient could not generate an appropriate response to the COVID vaccine as his regulatory cells are defective, or the COVID vaccine induced a mechanism similar to the cytokine storm. The patient tested negative for COVID inpatient, and thus the latter can be assumed. Interestingly, the patient was managed on low-dose steroids outpatient. Steroids act by reducing cytokine expression, lymphocyte differentiation, and proliferation [10]. If the patient, however, was not medically optimized, it is possible that the patient could have developed a cytokine storm, despite being on steroids. Additionally, up to 15% of patients may experience worsening MG symptoms and/or exacerbations upon initiation of oral prednisolone therapy [10].

Previous studies have compiled advice for clinicians managing patients with COVID and MG. During the beginning phases of the pandemic, literature suggested avoiding non-invasive ventilation as it can increase the risk of aerosolization of viral particles; however, this has since been disproven [8]. Immunosuppressant drugs and plasma exchange are highly recommended and safe in this patient population [1,5]. In fact, patients who were treated with prednisone and a second immunosuppressant drug were less likely to require mechanical ventilation in a previous study [5]. Some evidence suggests that tocilizumab is effective in MG exacerbations without the presence of COVID-19, which may have been helpful in our patient’s case as he was COVID-19-negative [8].

## Conclusions

In summary, we present a case of MG crisis secondary to COVID-19 vaccination. The patient tested negative for severe acute respiratory syndrome coronavirus 2 (SARS-CoV-2) but had symptoms such as fatigue, arthralgia, fever, chills, and dysphagia leading up to the presentation. This rare yet severe presentation that required intubation and mechanical ventilation in our patient raises the question of whether monitoring in a hospital setting is necessary for this subset of patients who get vaccinated. The usual post-vaccination observation time that lasts 15-30 minutes does not fully capture the adverse effects that may appear one or two weeks after the injection. In patients like this, the possibility of an MG flare and resultant respiratory muscle depression are life-threatening but avoidable if proper parameters are put in place for observation within the first and second weeks of receiving the vaccine. Further research must be done to identify the prevalence of this adverse outcome as well as to identify the timing of presentation in order to propose adequate observational measures. In the end, the risk of contracting SARS-CoV-2 infection and its consequences including acute respiratory failure, acute respiratory distress syndrome, lung fibrosis, hypercoagulability, and death outweigh the risk of adverse events from vaccination. Vaccination is still the preferred modality to combat the virus, and understanding the timing and prevalence of adverse events can help formulate the precautions needed to be taken during and after vaccine administration.
